# Novel Biomarkers of Gastric Cancer: Current Research and Future Perspectives

**DOI:** 10.3390/jcm12144646

**Published:** 2023-07-12

**Authors:** Yasushi Sato, Koichi Okamoto, Yutaka Kawano, Akinari Kasai, Tomoyuki Kawaguchi, Tamotsu Sagawa, Masahiro Sogabe, Hiroshi Miyamoto, Tetsuji Takayama

**Affiliations:** 1 Department of Community Medicine for Gastroenterology and Oncology, Tokushima University Graduate School of Medical Science, Tokushima 770-8503, Japan; 2 Department of Gastroenterology and Oncology, Tokushima University Graduate School of Medical Science, Tokushima 770-8503, Japan; okamoto.koichi@tokushima-u.ac.jp (K.O.); ykawano@tokushima-u.ac.jp (Y.K.); oneok824@icloud.com (A.K.); kawaguchi.tomoyuki@tokushima-u.ac.jp (T.K.); sogabe.masahiro@tokushima-u.ac.jp (M.S.); miyamoto.hiroshi@tokushima-u.ac.jp (H.M.); takayama@tokushima-u.ac.jp (T.T.); 3 Department of Gastroenterology, Hokkaido Cancer Center, Sapporo 060-0042, Japan; stamotsu@jk9.so-net.ne.jp

**Keywords:** gastric cancer, PD-L1, HER2, MSI, FGFR2, claudin 18.2, targeted therapy

## Abstract

Gastric cancer is a heterogeneous disease with diverse histological and genomic subtypes, making it difficult to demonstrate treatment efficacy in clinical trials. However, recent efforts have been made to identify molecular biomarkers with prognostic and predictive implications to better understand the broad heterogeneity of gastric cancer and develop effective targeted therapies for it. HER2 overexpression, HER2/neu amplification, MSI-H, and PD-L1+ are predictive biomarkers in gastric cancer, and a growing number of clinical trials based on novel biomarkers have demonstrated the efficacy of targeted therapies alone or in combination with conventional chemotherapy. Enrichment design clinical trials of targeted therapies against FGFR2b and claudin 18.2 have demonstrated efficacy in unresectable advanced gastric cancer. Nonetheless, it is essential to continuously validate promising molecular biomarkers and introduce them into clinical practice to optimize treatment selection and improve patient outcomes. In this review, we focused on established (PD-L1, HER2, MSI) and emerging biomarkers (FGFR2, CLDN18.2) in gastric cancer, their clinical significance, detection methods, limitations, and molecular agents that target these biomarkers.

## 1. Introduction

Gastric cancer (GC) is the fifth most common cancer worldwide, accounting for 5.6% of all new cancer cases (>1 million) in 2020. It is the fourth leading cause of cancer-related mortality, accounting for 7.7% of cancer deaths [1]. GC is more common in males and is particularly prevalent in Asia, with an incidence rate as high as 29.5/100,000 in Japan and South Korea [2]. The etiology of GC is still unknown, but chronic gastritis is thought to be responsible for cellular changes leading to malignant transformation; *Helicobacter pylori* infection is the strongest known risk factor for GC [3]. The incidence of GC has steadily decreased worldwide over the past 50 years due to the prevention and treatment of *H. pylori* infection, food preservation, and dietary changes [4]. On the other hand, an increase in proximal stomach and gastroesophageal junction (GEJ) cancers has been observed in association with obesity and poorly controlled gastroesophageal reflux disease (GERD) [5].

Surgery and chemotherapy remain the most preferred curative treatments for GC. Due to the early detection of GC, advances in chemotherapy and molecularly targeted therapies, and multidisciplinary treatment, the mortality rate has declined over the past few decades. Nevertheless, the overall survival rate for GC remains low, with a 5-year survival rate of approximately 20% worldwide, except in Japan and Korea, where screening programs for the early detection of gastric cancer are well-established [6]. Therefore, many current new strategies are aimed at detecting GC at an early stage, or even at an advanced stage, to optimize treatment through personalization.

In recent years, with the expansion of knowledge about tumor biology and biomarkers, cancer treatment has moved away from a “one-size-fits-all” approach using conventional cytotoxic chemotherapy agents to biomarker-driven, personalized therapies tailored to patient-specific characteristics (especially the molecular profile of the tumor). Biomarkers play an important role in the treatment of GC, as they help determine cancer treatment strategies and predict clinical outcomes [7].

Biomarkers are biological molecules in the blood, fluids, or tissues in the body that indicate normal or abnormal processes, conditions, or diseases, such as cancer; they can include a protein, nucleic acid, antibody, peptide, and many others [8]. They can be broadly classified into prognostic biomarkers, which predict cancer progression, and sensitivity-predictive biomarkers, which predict response to treatment [9]. The use of biomarkers is promising in the development of new anticancer agents because of their many advantages, including reduced toxicity, cost-effective use, and better patient selection for higher research and development success rates and lower development costs.

The development of molecularly targeted therapies has led to various clinical trial designs aimed at identifying subgroups of patients who are most likely to benefit from them. For example, large randomized controlled trials examining the efficacy of an intervention may include the “enrichment” design, in which “only biomarker-positive patients are included in the randomized controlled trial”; the “all-comers” design, in which “biomarkers are measured but all eligible patients are included in the randomized controlled trial regardless of outcome”; and the hybrid design, in which “biomarkers are measured and positive patients are randomized to the study treatment group and the standard treatment group, while negative patients are assigned to the standard treatment group [10]”.

Compared to colorectal and lung cancers, the development of biomarker-driven molecular-targeted therapeutics for GC has lagged behind. This is because GC is a heterogeneous disease with diverse histological and genomic subtypes, making it difficult to demonstrate treatment efficacy in clinical trials.

In contrast, beyond histopathological classification, substantial efforts have been made in recent years to characterize the genome of GC and identify molecular biomarkers with prognostic and predictive implications to better understand the broad heterogeneity of GC and to develop effective targeted therapies for it. For example, based on genomic and epigenomic alteration data, The Cancer Genome Atlas (TCGA) research program has proposed four molecular subtypes of GC: Epstein–Barr virus (EBV)-induced, microsatellite-unstable (MSI), genomically stable (GS), and chromosomal instability (CIN) [11]. These four subtypes differ in their clinicopathological and molecular biological features as well as in their tumor immune microenvironment [12,13].

As a result of a better understanding of the molecular profile of GC, new targets and agents are being discovered. New therapeutic strategies based on predictive biomarkers matched with therapeutic agents are being developed through a comprehensive understanding of the underlying targeting mechanisms [14]. HER2 overexpression and HER2/neu (ERBB2) amplification were the first molecular biomarkers identified to predict the efficacy of targeted therapies in GC, and microsatellite instability-high (MSI-H) and programmed death-ligand 1 positivity (PD-L1+) are established GC biomarkers. A growing number of clinical trials based on novel biomarkers have also demonstrated the efficacy of targeted therapies alone or in combination with conventional chemotherapy [14].

Many of the large randomized controlled trials for GC that are currently underway are being conducted with the enrichment design, and it is anticipated that biomarkers for predicting treatment response in GC will expand in the future. Phase III trials in enrichment design have shown the efficacy of bemarituzumab targeting FGFR2b [15] and zolbetuximab targeting claudin18.2 in unresectable advanced GC [16]. In these cases, the tissue expression of the target molecule of the antibody drug is a predictive biomarker of treatment response. Thus, it is increasingly important to validate promising molecular biomarkers and introduce them into clinical practice to select the optimal treatment for patients with GC and improve their prognosis. This review describes the major biomarkers in GC, their clinical significance, detection methods, limitations, and associated targeted agents. Special focus was given to conventional biomarkers for cytotoxic chemotherapy agents and established (HER2, PD-L1, MSI) and emerging molecular biomarkers, including fibroblast growth factor receptor 2 (FGFR2) and claudin 18.2 (CLDN18.2).

## 2. Biomarkers to Predict Responses to Cytotoxic Antitumor Drugs for Gastric Cancer

Cytotoxic antitumor agents are still the mainstay of GC treatment, including antimetabolites that act on DNA replication enzymes (e.g., fluorouracil), platinum agents that bind to and cross-link double-stranded DNA to inhibit DNA replication (e.g., cisplatin and oxaliplatin), topoisomerase inhibitors that suppress DNA recombination during cell division (e.g., irinotecan), and microtubule-stabilizing agents that bind to microtubules, inhibit spindle function, and prevent cell division (e.g., paclitaxel). Studies have been conducted on biomarkers predictive of the response to each anticancer agent by focusing on intracellular metabolic pathways and enzymes characteristic of the mechanism of action of each respective agent.

Extensive DNA damage involving dozens of base pairs is repaired by the nucleotide excision repair (NER) pathway. ERCC1, an enzyme that plays an important role in the NER pathway, binds to the DNA damage site and cleaves, removes, and repairs the damaged DNA. On the other hand, platinum-based drugs such as cisplatin (CDDP) induce cell death by forming cross-links within and between DNA strands. Since cross-linked adducts are suitable substrates for NER, the relationship between ERCC1 gene expression and CDDP sensitivity has attracted attention. Moreover, there have been several reports suggesting that ERCC1 expression is a useful predictor of response to anticancer therapy, including platinum-based drugs, in patients with advanced GC [17,18,19,20]. For example, it has been reported that high ERCC1 expression is associated with significant tumor reduction and prolonged survival in patients with GC receiving CDDP and fluorouracil (5-FU) (FP) [17]; 5-FU, folinic acid, and oxaliplatin (FOLFOX) therapy [18]; and docetaxel, CDDP, and S-1 (DCS) [20] as neoadjuvant chemotherapy regimens.

The antimetabolite drug 5-FU is phosphorylated by thymidine phosphorylase (TP) in the body and becomes fluorodeoxyuridine monophosphate (FdUMP), a metabolically active substance. FdUMP then binds to thymidylate synthase (TS), which is necessary for DNA synthesis, and forms a ternary complex under reduced folate conditions and inhibits TS activity, impeding DNA synthesis and cell growth. On the other hand, more than 85% of 5-FU is reduced to inactive metabolites by dihydropyrimidine dehydrogenase (DPD) in the liver and is excreted from the kidneys; DPD activity is closely related to 5-FU efficacy [21]. Among the enzymes associated with fluorouracil metabolism in GC tissues, TS and DPD have been reported to be useful markers [22,23,24,25]. However, the clinical utility of these biomarkers for cytotoxic antitumor agents has not been established because, unlike molecularly targeted drugs with high biomarker affinity, they do not specifically inhibit the target molecule and are mostly analyzed using small, retrospective samples.

## 3. Established Molecular Biomarkers in Gastric Cancer

### 3.1. Human Epidermal Growth Factor Receptor 2 (HER2)

#### 3.1.1. Molecular Characteristics of HER2

HER2 is a proto-oncogene encoded by *ERBB2* on chromosome 17 and is a membrane-bound receptor tyrosine kinase. It is one of four members of the human EGFR family, including EGFR/HER1, HER2/neu, HER3, and HER4, that are associated with tumor cell growth, adhesion, migration, and differentiation [26]. HER2 overexpression can further induce its homodimerization and heterodimerization with other members of the EGFR family, which can then activate signaling pathways leading to tumor growth [27]. In contrast to other members of the EGFR family, HER2 is constitutively active and can initiate signaling pathways without requiring a binding activator ligand.

#### 3.1.2. HER2 Expression in Gastric Cancer

HER2 is overexpressed in 5–25% of GC cases and approximately 20–32% of GEJ cancer cases. HER2 overexpression is most commonly caused by the amplification of the *HER2* gene [28,29], although constitutive HER2 activation can also occur due to gene fusion [30]. HER2 overexpression depends on the site (upper third of the stomach), CIN subtype, and degree of differentiation (well-differentiated or moderately differentiated). In histological evaluations, HER2 overexpression/amplification rates were more predominantly seen in intestinal than diffuse-type tumors [28,31,32,33].

**Figure 1:**
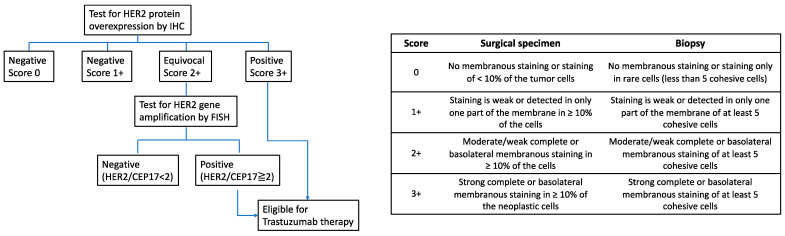
Human epidermal growth factor receptor 2 testing algorithm. Abbreviations: HER2: human epidermal growth factor receptor 2; IHC: immunohistochemistry; FISH: fluorescence in situ hybridization; CEP17: chromosome enumeration probe 17.

Immunohistochemistry (IHC) and in situ hybridization (ISH) are currently recommended to detect HER2 overexpression/amplification [34]. HER2 overexpression is defined as an IHC score of 3+; if HER2 staining is unclear (IHC 2+), ISH should be performed to confirm the amplification status of the HER2 gene [35]. ISH results showing a HER2/chromosome enumeration probe 17 (HER2/CEP17) ratio greater than 2 are considered positive [35] (Figure 1).

The main limitation of slide-based HER2 assays is the intratumor heterogeneity of HER2, which is common in GC and has been reported to be found in 33–52% of cases [36,37]. The high incidence of HER2 heterogeneity is considered the main reason for the high discordance rate (12.3%) between paired biopsy and resection specimens. This discordance has also been observed in 20% of paired primary and distant metastatic HER2+ GC cases [36]. Therefore, diagnosing HER2 positivity in GC requires multiple biopsy specimens (at least four), and HER2 IHC assays should be performed on both biopsy/resection specimens and primary/metastatic sites, even if one of them is negative [36,37].

In addition, the intratumor heterogeneity of HER2 expression can induce false-negative results for HER2 amplification via next-generation sequencing (NGS) [38]. An appropriate tumor heterogeneity index (THI; HER2 IHC H-score multiplied by the tumor volume) is necessary because a low THI affects the detection rate of HER2 amplification using NGS [39].

Approaches focused on liquid biopsy using readily available circulating tumor DNA (ctDNA) have also been investigated in gastric/gastroesophageal junction (G/GEJ) cancer clinical trials [39]. The advantages of such an approach may be its better suitability for sampling a comprehensive tumor profile and detecting HER2 status throughout the primary tumor and any metastases, compared to limited tissue sampling with biopsies restricted to specific locations within GC lesions [40]. For example, circulating tumor DNA (ctDNA) assays can assess whether HER2 gene amplification persists after trastuzumab treatment [41].

Many reports state that HER2 expression status per se (without HER2-related therapy) is not a prognostic factor [42,43] as it does not directly impact survival in patients with advanced GC. However, it is controversial as several retrospective studies suggest that HER2 expression is a negative prognostic factor associated with poorer survival outcomes [44,45].

#### 3.1.3. HER2-Targeted Therapy

HER2-targeted therapy, the first molecular-targeted agent approved as a standard therapy for GC, has dramatically improved the outcome of HER2-positive GC. Trastuzumab is a monoclonal antibody that targets the HER2 receptor, causing the downregulation of HER2 expression. In the Trastuzumab for Gastric Cancer (ToGA) trial, treatment with trastuzumab plus CDDP and fluoropyrimidine improved overall survival (OS) compared to chemotherapy alone in patients with advanced gastric or GEJ cancer with HER2 overexpression (13.8 months vs. 11.1 months; *p* = 0.005) [43]. Furthermore, the OS benefit of trastuzumab was demonstrated in patients with high HER2 expression (IHC 2+/FISH+ or IHC 3+) but not in patients with low HER2 expression (IHC 0 or 1+/FISH+) [43]. However, using several HER2-targeted agents other than trastuzumab (such as lapatinib, pertuzumab, or trastuzumab-emtansine) as primary or secondary therapies failed to improve the clinical outcome of patients with HER2+ GC [46]. The following underlying resistance mechanisms may contribute to the discouraging results of HER2-targeted therapy: (1) the intratumor heterogeneity of HER2, (2) the aberrant activation of the PIK3CA signaling pathway (downstream of HER2), and (3) the simultaneous amplification of *EGFR*, *MET*, and *CCNE1* [47]. In addition, the loss of HER2 positivity occurs after treatment with trastuzumab in approximately one-third of patients with HER2-positive advanced GC; this may be the mechanism promoting resistance to subsequent anti-HER2 therapy [48,49]. Indeed, the continued use of trastuzumab beyond progression (TBP) is not effective in patients with GC refractory to initial treatment with trastuzumab and chemotherapy [50]. Hence, the failure of the TBP strategy may be partly due to decreased HER2 positivity rather than an acquired resistance to trastuzumab [51].

In contrast, the DESTINY-Gastric01 trial showed that treatment with trastuzumab deruxtecan (T-DXd), a HER2-directed antibody–drug conjugate (ADC), resulted in a significant improvement in both the objective response rate (ORR) and OS for patients with HER2-positive (IHC 3+ or IHC 2+ with FISH+) locally advanced or metastatic G/GEJ cancer that had progressed after at least two previous regimens, including trastuzumab [52]. The high drug-to-antibody ratio and membrane permeability of the active payload of ADCs may explain their efficacy, leading to a bystander effect that is less dependent on HER2 tumor expression. This suggests that ADCs may be clinically effective even in cells with low or heterogeneous HER2 expression [52]. Preliminary evidence shows that T-DXd has clinical activity in an exploratory cohort of patients with low HER2 (IHC 2+/ISH− or IHC 1+) G/GEJ adenocarcinoma who did not receive anti-HER2 treatment in the DESTINY-Gastric01 trial [53].

Several novel HER2-targeted agents for GC are currently in development to overcome trastuzumab resistance, including bispecific antibodies (zanidatamab) [54,55], chimeric antibodies with augmented antibody-dependent cellular cytotoxicity (ADCC) (margetuximab) [56], and small molecule kinase inhibitors (afatinib, neratinib, and tucatinib) [57,58,59].

Zanidatamab, a bispecific anti-HER2 antibody that simultaneously binds two non-overlapping HER2 epitopes, is used to treat HER2-positive G/GEJ adenocarcinoma. A phase Ib/II trial is underway for zanidatamab in combination with the anti-PD-1 antibody tislelizumab, which was designed to minimize antibody-dependent phagocytosis by macrophages [60,61]. In addition, margetuximab is a HER2-targeting antibody with an engineered FCγ domain. It is designed to improve the involvement of the immune system in GC compared to other anti-HER2 therapies by enhancing binding to the activating Fc receptor FcγRIIIA (CD16A) and reducing binding to the inhibitory Fc receptor FcγRIIB (CD32B) through a specific modification in the Fc domain. It has demonstrated antitumor activity in combination with pembrolizumab in phase Ib/II trials [56]. HER2 therapy upregulates PDL1 expression; this resistance mechanism laid the foundation for combining it with immune checkpoint inhibitors (ICIs) [62]. In patients with unresectable or metastatic HER2-positive G/GEJ adenocarcinoma, pembrolizumab in combination with trastuzumab and chemotherapy significantly improved the ORR compared to placebo in combination with trastuzumab and chemotherapy, according to an interim analysis of the KEYNOTE-811 trial [63]. In the future, anti-HER2 agents are expected to overcome HER2 therapy resistance through their combination with ICIs and targeted agents against coexisting mutations and amplifications.

### 3.2. Biomarkers in the Treatment of ICIs in Advanced Gastric Cancer

PD-1/PD-L1 inhibitors are changing the treatment of advanced GC. However, not all patients can currently benefit from treatment with PD-1/PD-L1 inhibitors. Several immunobiological markers may help appropriately screen patients suitable for treatment with PD-1/PD-L1 inhibitors. In particular, biomarkers such as PD-L1, MSI-H, and TMB status have been reported to assess the efficacy of ICIs in patients with GC.

#### 3.2.1. PD-L1

##### Molecular Characteristics of PD-L1

PD-1 is an inhibitory checkpoint receptor protein expressed on cytotoxic T cells and other immune cells [64]. Specifically, PD-1 is expressed by activated T cells, tumor-infiltrating lymphocytes, and other immune cells in the germinal centers of lymphoid follicles [65]. By binding to its ligands PD-L1 and PD-L2, PD-1 maintains immune cell tolerance during peripheral and central immune regulation [64]. The binding of PD-L1 to PD-1 inhibits T cell receptor signaling. Some tumor cells express high levels of PD-L1 as an immune evasion mechanism since the PD-1/PD-L1 interaction induces the inactivation of cytotoxic T cells and the downregulation of immune responses [66]. In addition, PD-L1 expression is regulated by the tumor microenvironment. It is modulated by the transcriptional regulation of inflammatory signaling (such as interferon [IFN]-α, IFN-β, IFN-γ, and nuclear factor-kappa B [NF-κB]) and oncogenic signaling (phosphoinositide 3-kinase [PI3K] and NF-κB) [67].

##### PD-L1 Expression in Gastric Cancer

PD-L1 is expressed on both tumor cells and the immune stroma (such as lymphocytes and macrophages) across all stages and histologies of G/GEJ adenocarcinoma [68]. ICIs targeting PD-1 or PD-L1 inhibit PD-1/PD-L1 interactions and restore cancer cell-directed immune responses, thus creating a paradigm shift in therapeutic strategies for several solid tumors, including GC [69]. PD-L1 expression in GC is significantly more common in males with proximal gastric, unclassifiable, papillary, HER2-positive, Epstein–Barr--virus-positive, microsatellite-unstable, and PIK3CA-mutant GC. A high expression of PD-L1/PD-1 is associated with significantly better patient prognosis, and PD-L1 has been reported to be an independent prognostic factor for survival [70].

Investigation of PD-L1 protein expression to identify patients most likely to benefit from anti-PD-1/PD-L1 therapy is typically conducted using IHC [71] in both tumor cells and immune cells [72]. PD-L1 status is assessed immunohistochemically by applying a tumor proportion score (TPS; percentage of PD-L1-positive tumor cells) and a combined positive score (CPS; the number of PD-L1-stained cells [tumor cells, lymphocytes, and macrophages] divided by the total number of viable tumor cells and multiplied by 100). The maximum score is defined as CPS 100, although the CPS may exceed 100. Tumors are classified as PD-L1-positive if their CPS is 1 or higher; in G/GEJ cancers, 47.3–82.0% of patients across studies were PD-L1-positive [73,74,75,76].

Currently, four PD-L1 assays are used to detect PD-L1 protein expression. IHC 22C3 pharmDx (Agilent), PD-L1 IHC 28-8 pharmDx (Dako/Agilent), PD-L1 SP142 assay (Ventana/Roche), and the PD-L1 SP263 assay (Ventana/Roche) are commercially available. Each assay predicts the clinical efficacy of four immunotherapeutic agents (pembrolizumab, nivolumab, atezolizumab, and durvalumab, respectively). Although the 22C3 pharmDx (Agilent, Santa Clara, CA, USA) is the only companion diagnostic approved by the US Food and Drug Administration (FDA) for GC, recent studies have reported evidence of possible compatibility across different PD-L1 assays [77,78,79].

Several limitations of using PD-L1 as a biomarker affect its usefulness as a predictor of response to ICIs. One of these limitations is the intratumor heterogeneity of PD-L1 expression. In GC, PD-L1 expression has been shown to show high discordance rates between biopsies and surgically resected tissue [80,81]. Significant spatial heterogeneity between primary tumors and metastases (61% concordance rate) and temporal heterogeneity between tumors before and after chemotherapy (57–63% concordance rate) have also been reported [82]. Accurately diagnosing PD-L1 expression requires obtaining at least five GC biopsy samples [80,81]. Interobserver variability in the assessment of PD-L1 expression is also a major challenge. For example, high discrepancy rates have been reported in estimating PD-L1 expression, especially around the clinically important cutoff value of 1% [78]. To solve these problems, attempts to improve diagnostic performance using digital pathology and artificial intelligence are expected to be implemented [83].

##### PD-1-Targeted Therapy

Monoclonal antibodies against PD-1 (pembrolizumab and nivolumab) have shown efficacy in large clinical trials in patients with advanced GC. A meta-analysis of 17 phase III randomized clinical trials of ICI treatment in GC reported that the PD-L1 CPS was the second strongest predictive factor for ICI benefit following MSI-H [84]. In GC, a PD-L1 CPS ≥ 1 generally defines a PD-L1-positive tumor. However, in the pembrolizumab trials, a CPS ≥ 10 demonstrated an enhanced response to treatment, while the nivolumab trials used a CPS cutoff of 5 for the primary endpoint of OS and progression-free survival (PFS).

In the phase II KEYNOTE-059 study for patients with advanced G/GEJ adenocarcinoma at the third or later lines of treatment (*n* = 259), pembrolizumab treatment provided an ORR of 15.5% for patients with PD-L1-positive tumors (CPS ≥ 1), while it was 6.4% for those with CPS < 1 as determined via PD-L1 IHC 22C3 pharmDx [74]. In the phase III KEYNOTE-061 trial for patients with previously treated G/GEJ adenocarcinoma with a PD-L1 CPS ≥ 1, pembrolizumab did not significantly improve the median OS compared with paclitaxel (9.1 vs. 8.3 months, hazard ratio [HR]: 0.82, 95% CI: 0.66–1.03, *p* = 0.0421). However, patients with higher PD-L1 CPS (≥10) had a better treatment response compared to those who received paclitaxel (median OS: 10.4 months vs. 8.0 months, HR: 0.64, 95% CI 0.41–1.02), suggesting that the proportion of tumor cells expressing PD-L1 may influence the therapeutic effect of pembrolizumab [75]. In the first-line setting (KEYNOTE-062), pembrolizumab monotherapy showed non-inferiority to chemotherapy regarding OS in patients with CPS ≥ 1. On the other hand, pembrolizumab plus chemotherapy failed to demonstrate OS superiority to chemotherapy in patients with CPS ≥ 1 or CPS ≥ 10 [85]. Furthermore, an integrated analysis of PD-L1 CPS cut-offs from the KEYNOTE-059, KEYNOTE-061, and KEYNOTE-062 trials consistently demonstrated improved median OS, response rate, and durable response with pembrolizumab therapy across treatment lines in patients with CPS ≥ 10 [86].

KEYNOTE-811, a study of pembrolizumab plus trastuzumab plus chemotherapy in previously untreated patients with unresectable or metastatic HER2-positive GC (*n* = 264) stratified by PD-L1 expression (CPS ≥ 1 or CPS < 1), revealed that 84.1% of patients had tumors with a PD-L1 CPS ≥ 1. A 95% confidence interval overlap was observed between the CPS ≥ 1 and CPS < 1 subgroups, but a greater difference in ORR was observed in patients with CPS ≥ 1 [63].

The phase III CheckMate-649 trial evaluated the clinical outcomes of nivolumab plus chemotherapy versus chemotherapy alone in patients with advanced or metastatic G/GEJ cancer as a first-line therapy. In this study, the nivolumab plus chemotherapy arm demonstrated statistically significant improvements in both median OS and PFS in patients with PD-L1 CPS ≥ 5 (OS, HR: 0.71, 98.4% CI: 0.59–0.86, *p* < 0.0001; PFS, HR: 0.68, 98% CI 0.56–0.81, *p* < 0.0001). Furthermore, a significant OS benefit was observed in patients with a PD-L1 CPS ≥ 1 (HR: 0.77, 99.3% CI: 0.6–0.92; *p* = 0.0001). These results indicate that nivolumab provides greater efficacy and benefit in patients with high PD-L1 CPS [76]. Thus, the response to immunotherapy with prolonged OS tends to depend on the level of PD-L1 CPS positivity, as demonstrated in the CheckMate-649 study with nivolumab (better OS response with CPS ≥ 5 than with CPS ≥ 1) and the KEYNOTE-062/061 study with pembrolizumab (better OS response trend in patients with CPS ≥ 10 than with CPS ≥ 1).

On the other hand, the benefit of patients with low CPS scores (CPS ≤ 5 or ≤1) in randomized phase III trials (CheckMate-649, KEYNOTE-062, KEYNOTE-590) that compared chemotherapy with additional ICI treatment were analyzed using the Kaplan–Meier subtraction method [87]. The results confirmed that patients with low PD-L1 CPS scores (CPS 1-9 and CPS 1-4 subgroups) did not show significant benefit from the addition of ICI compared to standard chemotherapy in advanced esophagogastric cancer (EGC) [87]. Further study will be needed on using ICIs for patients with low PD-L1 CPS scores.

#### 3.2.2. MSI-H/dMMR

Since the publication of the pivotal molecular classification of gastric adenocarcinoma in 2014 [11], the MSI status has become a predictive marker in GC not only for prognosis but also for immune checkpoint inhibition.

##### Detection of MSI-H/dMMR

Microsatellites are short repeats of DNA units ranging from one to six bases in length that are widely distributed and mostly located near the coding regions. Polymorphisms and instability in microsatellite regions, commonly referred to as microsatellite instability (MSI), result from errors due to unequal crossing over and polymerase slippage during DNA replication and recombination. Over the past years, MSI has been extensively studied for its role in the development and progression of various cancers, especially colorectal cancer [88].

The MSI-H (high level of microsatellite instability) phenotype results from mutations in repetitive sequences due to defects in the DNA mismatch repair (MMR) system, which is essential for monitoring and correcting DNA replication errors [89]. MSI-H can occur within the context of inherited syndromes, such as Lynch syndrome, with germline mutations in MLH1, MSH2, MSH6, or PMS2, or sporadically through somatic mutations in the *MMR* gene. The epigenetic silencing of MLH1 via promoter hypermethylation is the primary mechanism leading to MMR loss in sporadic and familial MSI-H GC cases [90]. The presence of defective mismatch repair (dMMR) can be determined using IHC, which detects the loss of MMR protein expression due to mutations in the *MMR* gene using antibodies against MLH1, PMS2, MSH2, and MSH6 [91].

DNA-based techniques, such as PCR and NGS using the single nucleotide markers BAT-25, BAT-26, NR-21, NR-24, and NR-27, are used to detect MSI-H in the tumor genome [92]. MSI evaluation via PCR shows high concordance (>90%) with dMMR via IHC [93]. The percentage of MSI-H (8–25%) in patients with GC has been reported to vary considerably depending on the geography of the analyzed cohort (Asians vs. Caucasians), the heterogeneity of the tumor stage distribution, and the assay applied to detect the MSI status [11,94]. The frequency of MSI-H depends on the tumor stage and has been reported to be highest in the lymph node-negative stage (up to approximately 20%) and lowest in the metastatic stage (<5%) [95]. MSI-H GC was found to be associated with older age (>65 years), the female sex, tumor location in the middle and lower gastric body, less frequent lymph node metastasis, and a lower tendency to invade the serosal layer [96]. Typical histologic features are represented by a predominance of highly pleomorphic tumor cells organized in a specific growth pattern, association with mucinous gastric carcinoma or mucin-6 positivity, and marked lymphocyte infiltration [91,94,97]. Thus, MSI-H could be suspected through the histological phenotype; however, one-third of tumors do not show a specific phenotype and should be tested for MSI regardless of the histological phenotype.

##### Clinical Significance of MSI-H/dMMR

The significantly better outcome of MSI-H GC compared to microsatellite-stable (MSS) GC has led to a study on the need for neoadjuvant/perioperative and adjuvant chemotherapy in MSI-H GC. An exploratory analysis of the MAGIC trial showed that patients with non-metastatic MSI-H GC have a better prognosis after surgery than those with MSS GC. However, patients with MSI-H GC had worse prognoses when treated with perioperative chemotherapy (median OS: 9.6 months vs. 19.5 months, HR: 2.18) [93]. Subgroup analyses showed no survival benefit with perioperative chemotherapy compared to surgery alone for patients with MSI-H EGC (meta-analysis of the MAGIC, CLASSIC, ARTIST, and ITACA-S trials) [98]. Based on these results, MSI or dMMR determined using preoperative biopsy may be used to select patients for perioperative chemotherapy. However, since these are retrospective studies, these results need to be validated using a large prospective study.

Preliminary data from the DANTE trial that compared perioperative atezolizumab with 5-fluorouracil, leucovorin, oxaliplatin, and docetaxel (FLOT) versus FLOT alone for resectable EGC showed benefit for patients with resectable MSI-H GC treated with ICI plus chemotherapy. The rate of pathologic complete response (pCR) or sub-total recovery (TRG1a/b) was 80% (8/10) in patients treated with FLOT plus atezolizumab and 59% (7/12) in those treated with FLOT alone. Unlike previous retrospective studies, this study supported the use of perioperative treatment for patients with MSI-H and G/GEJ adenocarcinoma, as patients with MSI-H had a very high rate of disease regression with FLOT or FLOT/atezolizumab [99]. In addition, the GERCOR NEONIPIGA phase II trial tested the efficacy of neoadjuvant nivolumab plus ipilimumab and adjuvant nivolumab in localized MSI-H/dMMR EGC. The primary endpoint was the pCR rate. According to their preliminary data, the pCR rate was 59% (17/29 patients), and 94% (30/32 patients) were event-free after 12 months of follow-up. Hence, neoadjuvant therapy with nivolumab and ipilimumab was feasible and associated with high pCR rates [100]. These results suggest that immunotherapy may replace surgery in patients with MSI-high/dMMR tumors. It is hoped that future large prospective studies will confirm the efficacy of perioperative immunotherapy in patients with MSI-H/dMMR tumors.

MSI-H/dMMR may also serve as a predictive biomarker for treatment with ICIs. For instance, significant treatment response was observed in previously treated patients (*n* = 27) with MSI-H advanced G/GEJ cancer regardless of PD-L1 CPS enrolled in the KEYNOTE-061 trial [75]. An exploratory analysis of KEYNOTE-062 data in patients with untreated advanced G/GEJ cancer showed a survival benefit with pembrolizumab compared with chemotherapy in a subgroup of patients whose tumors were MSI-H and PD-L1 CPS ≥ 1 (*n* = 33; median OS: not reached vs. 8.5 months; HR: 0.29; 95% CI: 0.11–0.81) [85]. In a meta-analysis of randomized trials involving treatment with or without PD-1 inhibitors in patients with advanced GC (KEYNOTE-062, CheckMate-649, JAVELIN Gastric 100, and KEYNOTE-061), 4.8% of patients with GC had MSI-H cancers [101]. The HR for OS benefit with anti-PD-1-based regimens was 0.85 (95% CI: 0.71–1.00) for MSS versus 0.34 (95% CI: 0.21–0.54) for MSI-H cancer. The treatment effect significantly differed between these two subgroups (P for interaction = 0.003). In the MSI-H subgroup, the HR for PFS was 0.57 (95% CI: 0.33–0.97; *p* = 0.04), and the odds ratio for treatment response was 1.76 (95% CI: 1.10–2.83; *p* = 0.02). This suggests that MSI-high patients are highly sensitive to PD-1/PD-L1-targeted immunotherapy [101]. Similarly, for nivolumab, a post hoc analysis of CheckMate-649 data showed that nivolumab plus chemotherapy for MSI-H G/GEJ cancer has clinically significant advantages over chemotherapy in terms of OS (median, 38.7 months vs. 12.3 months, HR: 0.38; 95% CI: 0.17–0.84) and ORR (55% vs. 39%) [102]. It was assumed that this was due to the increased abundance of immunogenic peptides/proteins (neoantigens) resulting from dMMR, some of which are processed and presented to HLA molecules on the tumor cell surface, making them more easily recognized by the immune system [103,104].

The FDA has approved pembrolizumab for patients with unresectable or metastatic MSI-H or dMMR solid tumors that have progressed after prior therapy and for patients with no alternative treatment options. The Japanese Pharmaceuticals and Medical Devices Agency has approved pembrolizumab for patients with advanced or recurrent MSI-H solid tumors that have progressed after chemotherapy and are refractory or intolerant to standard therapy, provided that the MSI-IVD kit (FALCO) companion diagnosis of MSI-H is demonstrated.

#### 3.2.3. Tumor Mutational Burden

Tumor mutational burden (TMB) is broadly defined as the number of somatic mutations per megabase of an interrogated genomic sequence. Tumors with high mutation burden (TMB-high, TMB-H) may promote the generation of immunogenic neopeptides on tumor cells. These tumor-specific neoantigens may promote tumor-specific T cell-mediated antitumor immunity and improve the efficacy of ICI therapy [105].

##### Clinical Significance of TMB

The KEYNOTE-158 trial in patients with previously treated unresectable or metastatic solid tumors showed that TMB-H (≥10 mut/Mb) was associated with clinically relevant improvements in pembrolizumab efficacy and may be a useful response-predictive biomarker [106]. Based on these data, the FDA approved pembrolizumab monotherapy for a subgroup of patients with solid tumors with TMB ≥ 10 mut/Mb. Although the data from the KEYNOTE-158 trial demonstrated the role of TMB in patient selection for cancer treatment, important issues remain, including the selection of an appropriate TMB cutoff.

##### Assessment of TMB

Measuring TMB requires a comprehensive genomic profiling assay applying an extensive multigene panel (typically covering regions > 1 Mb). The prevalence of tissue TMB-H using a cutoff value of 10 mut/Mb has been reported in 13–17% of samples in randomized phase III trials in patients with G/GEJ cancer [106,107]. Besides whole-exome sequencing (WES) analysis of tumor tissue, there are less invasive TMB measurements using panel-based NGS in ctDNA isolated from blood [108,109].

The association between tissue TMB (WES cutoff: 175 mutations/exome) and response to pembrolizumab was reported in the KEYNOTE-061 trial, an exploratory analysis of 420 patients with GC comparing the effects of second-line pembrolizumab therapy to chemotherapy [107]. The ORR for patients with TMB ≥ 175 mutations/exome was 30%, the ORR for patients with TMB < 175 mutations/exome was 11%, and the median OS for both cohorts was 16.4 months and 8.1 months, respectively (HR: 0.46; 95% CI: 0.27–0.81). Furthermore, even after excluding MSI-H patients from this analysis, tissue TMB correlated with improved outcomes upon pembrolizumab treatment, suggesting that tissue TMB may be an independent predictor of response to ICI treatment [107]. Similar results were reported when tissue TMB was determined using a panel-based technique with a 10 mut/Mb cutoff (*n* = 205). The strong association between tissue TMB and pembrolizumab efficacy suggests that tissue TMB is an important and independent predictor beyond PD-L1 status [107]. Based on these results, pembrolizumab and the companion diagnostic assay, FoundationOne CDx™ (Foundation Medicine, Cambridge, MA, USA), are approved by the FDA for TMB-H (≥10 mut/Mb) patients with second-line unresectable or metastatic solid tumors.

As the use of TMB as a predictive biomarker in clinical practice increases, several commercial and laboratory-developed test panels are currently under development. However, depending on the platform used, the outcomes of panel TMBs vary, with some panels consistently overestimating or underestimating TMB. The size and composition of the panels and the developing institute’s bioinformatics algorithms, such as the type of mutations and variant filters used to calculate TMB, may have contributed to these differences. Therefore, efforts have been initiated to improve the consistency and reliability of panel TMB estimates, including the standardization of TMB reporting [110].

Regarding the association between PD-L1 expression and TMB, it is reasonable to assume that a higher TMB would induce a higher density of neoantigen-specific tumor-infiltrating lymphocytes, leading to IFN-γ secretion and the upregulation of PD-L1 expression in tumor cells [111]. However, in most cancer subtypes, only a slight association was shown at the tumor sample level (Pearson’s correlation coefficient: 0.084) [104]. This suggests that PD-L1 and TMB are independent predictive biomarkers that may contribute to identifying patients suitable for immune checkpoint therapy. Interestingly, among all examined carcinomas, the association between PD-L1 expression and TMB was reported to be the strongest in GC (Pearson’s correlation coefficient > 0.3) [104].

Genomic signatures contributing to TMB-H in MSS gastrointestinal tumors have been reported [112]. Gene mutations that attenuate or activate the tumor immune response despite being TMB-H have been used to construct and propose a modified TMB (mTMB) prediction model for ICI efficacy. When comparing the efficacy of ICI in patients with and without mutations in this mTMB gene signature, patients with mutations in the mTMB gene signature showed a significant benefit with ICI, with an HR of 0.55 (95% CI: 0.31–0.99). It should be noted that not all mutations related to TMB-H can enhance antitumor immune response. Developing more composite biomarkers, such as the mTMB signature, is important for better patient selection for ICI treatment.

#### 3.2.4. Immune-Related Adverse Events

Along with robust antitumor activity, ICI therapy also exhibits nonspecific systemic effects due to immune activation. Common immune-related adverse events (irAEs) observed in patients with GC undergoing treatment with nivolumab include diarrhea/colitis, hyperglycemia, adrenal insufficiency, and peripheral motor neuropathy [76,102]. Hence, it is crucial for healthcare providers to closely monitor and manage these irAEs to ensure patient safety and optimize treatment outcomes. Additionally, the relationship between irAEs and antitumor responses in cancer patients treated with ICIs has garnered interest [113]. However, limited research has been conducted on the correlation between irAEs and nivolumab efficacy in advanced GC. Masuda et al. conducted a retrospective study involving GC patients treated with nivolumab monotherapy, and their findings demonstrated that the development of irAEs was associated with improved clinical outcomes [114]. In the irAE group, the median PFS was 7.5 months, compared to 1.4 months in the non-irAE group (HR  =  0.11, *p* < 0.001). Furthermore, the median OS was 16.8 months in the irAE group, while it was only 3.2 months in the non-irAE group (HR  =  0.17, *p* < 0.001). These findings suggest that irAEs may serve as positive prognostic markers in GC patients undergoing nivolumab treatment. However, further research is needed to understand the underlying mechanisms and patient characteristics associated with these events, which may lead to the identification of promising ICI biomarkers.

## 4. Emerging Biomarkers

### 4.1. Fibroblast Growth Factor Receptor 2 (FGFR2)

#### 4.1.1. Molecular Characteristics of FGFR2

The fibroblast growth factor receptor (FGFR), a transmembrane tyrosine kinase receptor, is a member of a protein family that drives many downstream pathways, including the mitogen-activated protein kinase (MAPK) and AKT pathways, which are essential for cell growth, survival, and migration [115]. The activation of FGFR signaling can be caused by gene amplification, activating mutations, and chromosomal translocations/fusions. Specifically, *FGFR2* gene amplification is known to be a poor prognostic factor in patients with metastatic GC, and a high copy number (≥30) identified via NGS-based genomic profiling is significantly associated with shorter PFS and OS [116].

#### 4.1.2. Assessment of FGFR2 Status

The prevalence of *FGFR2* amplification is rare in GC; it varies by country, ranging from 4–7.4% in previous studies [115,116]. *FGFR2* amplification is significantly associated with lymph node metastasis, with 24% of *FGFR2*-amplified cases showing intratumoral heterogeneity [117]. So far, there is no established association between *FGFR2* amplification and gender, anatomical site, histological subtype, or TNM classification [118]. In contrast, a study using IHC to determine FGFR2 protein expression reported that FGFR2 was overexpressed in up to 60% of patients with GC [119]. A meta-analysis found that FGFR2 protein overexpression was associated with deeper tumor invasion, higher rates of lymph node metastasis, more advanced stages, and worse outcomes [119].

Detection of FGFR2 amplification at the molecular level using ISH or NGS is problematic due to the low frequency of FGFR2 amplification and the time and expense involved as a screening method. IHC staining for FGFR2 isoform IIb (FGFR2b) strongly correlates with FGFR2 copy number changes with high sensitivity and specificity [120], making IHC for FGFR2b a rapid and efficient screening tool to stratify patients with GC who may benefit from FGFR2-targeted therapy [120]. Positive FGFR2b expression was assessed via membranous and cytoplasmic staining. In addition, the overexpression of FGFR2b and higher H-scores in IHC are significantly associated with N-stage progression and shorter survival, which is consistent with the results of *FGFR2* gene amplification [120]. Interestingly, analysis of matched primary GC and metastatic GC tissue showed more FGFR2b+ tumors in metastatic sites than in the primary tumor. Moreover, the overexpression of FGFR2b was also frequently seen in metastatic lymph node tissue; therefore, both primary and metastatic gastric cancer tissue should be examined to select patients with GC for treatment with FGFR2 inhibitors [120]. Collectively, these findings suggest that *FGFR2* amplification may play an important role in tumor progression in GC, particularly in lymphatic metastasis.

#### 4.1.3. FGFR2-Targeted Therapy

A novel FGFR2 inhibitor, bemarituzumab, is a first-in-class, afucosylated, humanized anti-FGFR2b monoclonal IgG1 antibody. It has shown promising results in GC patients with *FGFR2* gene amplification in the FIGHT study, a phase II clinical trial [121,122]. The FIGHT study included patients with locally advanced or metastatic GC who were HER2-negative and had FGFR2b overexpression (either 2+ or 3+ staining, as detected via IHC) or *FGFR2* amplification (ctDNA) and received mFOLFOX6 plus bemarituzumab as first-line therapy. The survival outcomes of the included patients were compared to the mFOLFOX6 plus placebo group. Thirty percent of patients whose tumors were evaluated were FGFR2b-positive. The primary endpoint, median PFS, was extended to 9.5 months in the bemarituzumab group versus 7.4 months in the placebo group (HR: 0.68, 95% CI 0.44–1.04; *p* = 0.073). The median OS was not reached (NR) in the bemarituzumab group and was reached after 12.9 months in the placebo group (HR: 0.58, 95% CI: 0.35–0.95; *p* = 0.027). Interestingly, the median PFS in the subset with ≥10% FGFR2b positivity was 14.1 months for bemarituzumab vs. 7.3 months for the placebo (HR: 0.44, 95% CI: 0.25–0.77) and the median OS was NR for bemarituzumab vs. 11.1 months for the placebo (HR: 0.41, 95% CI: 0.22–0.79). This finding indicates that the higher the percentage of tumor cells expressing FGFR2b, the more effective the targeted therapy. Furthermore, it should be noted that bemarituzumab is beneficial regardless of *FGFR2* gene amplification status. Therefore, detection methods at the molecular level may become less important in future studies. Based on these positive results, a validating phase III trial in GC overexpressing FGFR2b (FORTITUDE-101:) and a phase Ib/III trial combining mFOLFOX6 plus nivolumab and bemarituzumab (FORTITUDE-102) is underway [123].

### 4.2. Claudin 18.2

#### 4.2.1. Molecular Characteristics of Claudin 18.2

Claudin family proteins comprise one of the major components of the tight junction complex [124]. Aberrant tissue expression of claudin proteins leads to the dysfunction of tight junctions, affecting various cell signaling pathways, and may contribute to the tumorigenic progression of some epithelial cancers [125]. CLDN18.2, a member of the claudin family, is predominantly present in the gastric mucosa and is retained even upon malignant transformation. Furthermore, it may become more exposed and accessible in malignant tissues as tight junctions are disrupted; hence, CLDN18.2 has attracted attention as a potentially attractive target for cancer therapy [126,127]

#### 4.2.2. Clinical Significance of Claudin 18.2

CLDN18.2 positivity has been reported in 30–33% of patients with G/GEJ cancer and is associated with a diffuse type of histology [128]. However, the clinicopathologic characteristics of CLDN18.2-positive G/GEJ cancer and its impact on treatment outcomes with current standard chemotherapy and anti-PD-1 therapy are unknown. Recently, Kubota et al. found that among patients with G/GEJ cancer, CLDN18.2 positivity (defined as moderate to strong CLDN18.2 expression in ≥75% of tumor cells) was identified in 24.0%, and CLDN18.2-positive tumors were associated with Borrmann type 4, *KRAS* amplification, low CD16, and high CD68 expression [129]. Meanwhile, the various molecular subtypes, such as dMMR, EBV-positive, HER2-positive, all-negative, and the CPS subgroup, showed nearly identical distributions. However, CLDN18.2 was not a predictor of response to chemotherapy or checkpoint inhibition. This suggests that CLDN18.2 may be targeted regardless of molecular subtype and may be useful when considering treatment strategies for CLDN18.2-positive G/GEJ cancer.

#### 4.2.3. Claudin 18.2-Targeted Therapy

Zolbetuximab is a chimeric IgG1 monoclonal antibody that binds to CLDN18.2 on the tumor cell surface and stimulates cellular and soluble immune effectors that activate ADCC and complement-dependent cytotoxicity [130]. The single-arm MONO clinical trial demonstrated a manageable safety profile and that 9% of patients with locally advanced/metastatic GC with CLDN18.2 expression achieved satisfactory efficacy with zolbetuximab monotherapy [130].

In the randomized phase II FAST trial, zolbetuximab in combination with the primary chemotherapy regimen epirubicin, oxaliplatin, and capecitabine (EOX) was associated with significantly better PFS (HR: 0.44, 95% CI: 0.29–0.67; *p* < 0.0005) and OS (HR: 0.55, 95% CI: 0.39–0.77; *p* < 0.0005) compared to EOX alone in G/GEJ cancer patients with moderate to strong CLDN18.2 expression in at least 40% of tumor cells. This significant PFS benefit was maintained even in patients with moderate to strong CLDN18.2 expression in >70% of tumor cells (HR: 0.38, 95% CI: 0.23–0.62) [16]. These results led to two phase III trials (SPOTLIGHT and GLOW) in patients with GC/GEJC with CLDN18.2 positivity (moderate to strong expression in >75% of tumor cells). The SPOTLIGHT trial is an international, double-blind, phase III study designed to evaluate the efficacy of modified FOLFOX6 (mFOLFOX6) plus zolbetuximab compared to mFOLFOX6 plus placebo in advanced G/GEJ cancer with positive CLDN18.2 expression (defined as ≥75% of tumor cells showing moderate to strong membranous CLDN18.2 staining) [131]. Both the primary (PFS) and secondary endpoints (OS) were significantly improved with mFOLFOX6 + zolbetuximab. The GLOW trial is a phase III study comparing zolbetuximab + CAPOX (capecitabine + oxaliplatin) with placebo + CAPOX as a first-line therapy in patients with Claudin18.2-positive, HER2-negative, advanced G/GEJ cancer. Zolbetuximab + CAPOX was associated with a 31.3% reduction in the risk of disease progression or death and a statistically significant increase in PFS compared to placebo + CAPOX, with a median PFS of 8.21 and 6.80 months, respectively. The OS also showed a statistically significant increase, with a 22.9% reduction in the risk of death. The median OS was 14.39 and 12.16 months for the zolbetuximab + CAPOX and placebo + CAPOX arms, respectively [132].

The criteria for Claudin18.2 positivity have not been sufficiently defined, mainly because the antibodies, staining, and scoring systems vary across published studies. These and future studies are expected to establish the optimal cutoff values at which antibody drugs are most effective.

## 5. Future Perspectives

Rapid advances in the molecular biology of tumors have led to significant advances in personalized medicine based on predictive biomarkers of susceptibility in GC treatment. However, considerable challenges remain in attaining ideal therapeutic outcomes. Trastuzumab is the standard treatment choice for advanced GC patients with HER2 overexpression, but HER2 is expressed in less than 20% of GC cases, and issues such as tumor heterogeneity and resistance to trastuzumab still need to be addressed. To overcome these challenges, new therapeutic agents currently under investigation, such as ADCs and HER2-targeted bispecific antibodies, have shown promise. Immunotherapy for PD-1 and MSI-H gastric cancer will also become the new standard of care for advanced GC, and combined immunotherapy with other targeted therapies to further enhance efficacy is expected to become widespread. So far, patient selection using PD-L1 scores has been considered for ICI therapy, but the appropriate threshold for PD-L1 positivity is still debatable. Establishing a new biomarker for immunotherapy would improve patient selection for ICI therapy and optimize patient response. In addition, the biomarker results may not necessarily reflect the clinical status of the patient during the treatment period, as biomarker expression may change over time and with selective post-treatment pressure. Moreover, biomarker results may differ depending on the biopsy site and whether the cancer is metastatic or primary. Therefore, it is also necessary to study patient treatment algorithms by considering cutoff values that consider the temporal and spatial heterogeneity of biomarkers.

By analyzing mutations and other genomic alterations in ctDNA derived from tumor cells and released into plasma, liquid biopsy has emerged as a non-invasive method to detect microresidual cancers to comprehensively profile the tumor genomic landscape for possible therapeutic targets. It can also be used to monitor the tumor genomic landscape over time for treatment failure or the appearance of treatment-resistant tumor subclones. Although liquid biopsy has not yet been used in the routine clinical management of patients with C/GEJ cancer, several cases of efficacy have been reported [133]. To predict the effect of trastuzumab on HER2-positive GC, ctDNA profiling based on liquid biopsy has been shown to be useful in monitoring the development of trastuzumab resistance and elucidating potential molecular mechanisms [134]. Given the invasiveness of tissue acquisition in patients, the clinical and genetic evaluation of malignant lesions may not be repeated. Liquid biopsy has the potential to provide informative data that can be obtained repeatedly instead of analyzing tumor tissue.

Measuring ctDNA as molecular residual disease may predict the risk of cancer recurrence after resection. Indeed, the association between ctDNA levels and clinical outcomes in postoperative patients with resectable GC has been evaluated, with ctDNA positivity predicting recurrence and association with worse disease-free survival and OS and preceding radiographic recurrence by a median of 6 months [135].

In a ctDNA-based observational screening study for patients with advanced gastrointestinal carcinoma, a direct comparison between liquid biopsy-based sequencing and tumor tissue-based sequencing was reported [136]. Compared to tissue-based genotyping, ctDNA genotyping significantly reduced screening time (11 vs. 33 days, *p* < 0.0001) and nearly doubled study enrolment rates (9.5 vs. 4.1%, *p* < 0.0001) without compromising treatment efficacy. This study shows that ctDNA profiling can reliably select patients for therapies matched to oncogenic drivers with clinical efficacies similar to tumor biopsy.

**Figure 2:**
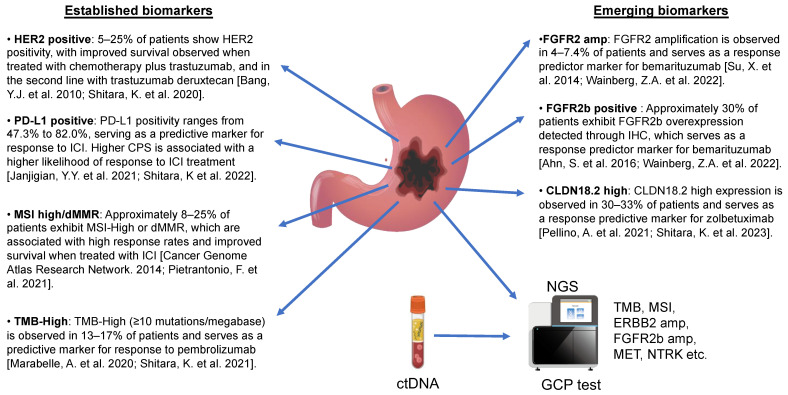
Key biomarkers tested in advanced gastroesophageal adenocarcinomas. Biomarkers are assessed via immunohistochemistry (IHC) in gastric cancer tissue for HER2 testing, PD-L1 CPS testing, and MMR evaluation. MSI and TMB are measured using polymerase chain reaction (PCR) or DNA-based assays such as comprehensive genomic profiling (CGP). CLDN18.2 and FGFR2b are novel biomarkers that utilize the IHC platform. CGP tests can detect various types of genomic alterations, including mutations, copy number variations, and structural rearrangements. They can be performed on different types of samples, including blood and tumor tissues, and analyzed using next-generation sequencing (NGS). Bang, Y.J. et al. 2010 [43], Shitara, K. et al. 2020 [52], Janjigian, Y.Y. et al. 2021 [63], Shitara, K et al. 2022 [76], Cancer Genome Atlas Research Network. 2014 [11], Pietrantonio, F. et al. 2021 [101], Marabelle, A. et al. 2020 [106], Shitara, K. et al. 2021 [107], Su, X. et al. 2014 [117], Wainberg, Z.A. et al. 2022 [122], Ahn, S. et al. 2016 [120], Wainberg, Z.A. et al. 2022 [122], Pellino, A. et al. 2021 [128], Shitara, K. et al. 2023 [131]. Abbreviations: amp, amplification; CPS, combined positive score; EGFR, epidermal growth factor receptor; ERBB2, Erb-B2 Receptor Tyrosine Kinase; FGFR2b, fibroblast growth factor receptor 2b; HER2, human epidermal growth factor receptor 2; ICI, immune checkpoint inhibitor; IHC, immunohistochemistry; MET, MET proto-oncogene, receptor tyrosine kinase; MMR, mismatch repair; MSI, microsatellite instability; NTRK, neurotrophic receptor tyrosine kinase; PD-L1, programmed death-ligand 1; TMB, tumor mutation burden.

Although diagnostic tissue samples are usually limited in GC, IHC, and genetic testing for the expression of MMR, PD-L1, FGFR2b, HER2, and CLDN18.2 will still be performed to determine the optimal treatment in each positive patient subgroup in the foreseeable future (Figure 2). Cancer is caused by genetic alterations, and additional genetic abnormalities accumulate during cancer development. Therefore, individual cancers may have different genetic alterations within the same histology of the same organ. The information obtained from cancer panel analysis, which can comprehensively analyze cancer-related gene alterations via comprehensive genome profiling using NGS, is an important biomarker for personalized cancer treatment [137].

Further progress in biomarker research is expected to lead to the accurate implementation and standardization of biomarker testing and the development of personalized medicine. Hence, more research on methods that reflect comprehensive and dynamic genetic conditions through rapid, simple, non-invasive, and repeatable liquid biopsy is warranted.

## 6. Conclusions

Recent advances in the molecular characterization of GC have enabled the development of targeted therapies and immunotherapies that significantly improve patient prognosis over conventional cytotoxic chemotherapy. Identifying novel biomarkers for GC is a promising area of research that can potentially improve diagnostic accuracy and patient outcomes. In addition, molecular profiling studies elucidated the molecular genetics of GC and helped develop new biomarkers and diagnostics, leading to the effective stratification of patients and the development of personalized clinical treatment using targeted therapies. Currently, only three biomarkers are used in routine practice to select patients with advanced stage G/GEJ cancer before administering molecularly targeted therapies: HER2 positivity for trastuzumab and T-DXd treatment, and MSI status and PD-L1 expression for anti-PD-1 treatment. With future improvements in sequencing technology and blood-based assay accuracy, biomarker testing is expected to be further integrated into the clinical practice of GC treatment. However, more studies are needed to validate their clinical utility and establish their role in clinical practice.

## Data Availability

Not applicable.
